# Non-Valvular Atrial Fibrillation in Young Adults in Eastern India: A Clinico-Aetiological Retrospective Analysis in a Tertiary Care Hospital

**DOI:** 10.7759/cureus.36918

**Published:** 2023-03-30

**Authors:** Satyapriya Mohanty, Anindya Banerjee, Abhinav Kumar, Pranjit Deb, Humshika Samantray, Debasish Das

**Affiliations:** 1 Cardiothoracic Surgery, All India Institute of Medical Sciences (AIIMS) Bhubaneswar, Bhubaneswar, IND; 2 Cardiology, All India Institute of Medical Sciences (AIIMS) Bhubaneswar, Bhubaneswar, IND

**Keywords:** retrospective analysis, eastern india, adults, young, nonvalvular atrial fibrillation

## Abstract

Background: The development of atrial fibrillation adds a lot to the morbidity and mortality of individual patients. The spectrum of non-valvular atrial fibrillation among young adults is less known. The present observational study aims to analyze the clinical-aetiological spectrum of non-valvular atrial fibrillation among young adults between 18 and 40 years of age.

Methods: A retrospective observational study was carried out to analyze the clinical-aetiological spectrum of non-valvular atrial fibrillation among young adults less than 40 years of age attending the cardiac outpatient department in a tertiary care hospital in Eastern India over a period of two years. Patients with any form of organic valvular heart disease and patients more than 40 years of age were excluded from the study. One hundred and seventeen patients under 40 years of age were analysed with respect to demographic, aetiological, and clinical profiles.

Results: Most common aetiologies behind non-valvular atrial fibrillation in young adults (<40 years) were hypertension (40%) and the presence of left ventricular systolic dysfunction (31%). Thyrotoxicosis, obesity, obstructive sleep apnoea, the presence of congenital heart disease, coronary artery disease, myopericarditis, chronic kidney disease, dyselectronemia, diabetes mellitus, and the presence of chronic obstructive pulmonary disease contributed towards the development of non-valvular atrial fibrillation in the young population in less proportion of cases. Most of the cases were symptomatic with palpitation, shortness of breath, or diaphoresis. Less number of cases (17%) had left atrial thrombus which may be due to early clinical attention with the proper therapeutic anticoagulation regimen.

Conclusions: Hypertension and the presence of left ventricular systolic dysfunction contribute to the majority towards the development of non-valvular atrial fibrillation among young adults. Accurate measurement and monitoring of blood pressure among young adults and careful assessment of left ventricular systolic dysfunction with subsequent appropriate management of hypertension and left ventricular systolic dysfunction in young can decrease the burden of non-valvular atrial fibrillation among the young population.

## Introduction

Atrial fibrillation is considered an old-age phenomenon among practising physicians and general people. The burden of non-valvular atrial fibrillation in the real-world scenario is yet unknown as most patients with atrial fibrillation present late unless they suffer from an episode of fast ventricular rate or heart failure. The profile and spectrum of non-valvular atrial fibrillation among relatively young adults are yet unknown. Early recognition of atrial fibrillation in young adults can save them from the devastating morbidity and mortality of young adults with stroke [1] which may be central or peripheral. Early onset of atrial fibrillation decreases exercise capacity [2], results in early-onset heart failure, and carries a significant risk of stroke in the future with additional morbidity and mortality. In the present study, we analysed the demography, aetiology, and clinical spectrum of non-valvular atrial fibrillation in young adults which will provide new insight among young practising physicians to be aware of the rhythm always if at all the patient presents with exertional or rest dyspnoea without a history of typical angina. Atrial fibrillation in the young most often occurs due to abnormal firing of the pulmonary veins [3] with anisotropic conduction across the atrium due to atrial fibrotic remodelling. Dilatation of the atrium, fibrotic remodelling, and increase in the atrial pressure together contribute towards the development of non-valvular atrial fibrillation in the young where multiple atrial wavelets with inhomogeneous conduction give rise to atrial fibrillation.

## Materials and methods

Aim and objective

The aim of this study was to retrospectively study and analyze the aetiology and clinical profile of young adults with non-valvular atrial fibrillation in a tertiary care centre in Eastern India.

Inclusion criteria

Patients between 18 and 40 years of age with non-valvular atrial fibrillation were included.

Exclusion criteria

Patients with any form of organic valvular heart disease (mitral, tricuspid, or aortic), end-stage organ disease (chronic liver disease, chronic kidney disease), malignancy, sepsis, and not willing to participate in the study were excluded.

The study was conducted in a tertiary care centre in Eastern India between May 2020 and 2022. Sample size calculation was done using the standard formula with the power of the study being 80% and an attrition rate of 15%. After obtaining the institutional ethical clearance from the institutional ethics committee (IEC) of All India Institute of Medical Sciences (AIIMS), Bhubaneswar, India (Ref: TE/IM-NF/CTVS/22) for retrospective analysis, we analysed the data of 117 patients attending the outpatient department of Cardiology and Cardiothoracic and Vascular surgery between May 2020 and 2022. Informed telephonic oral patient consent was obtained from each of those patients prior to analysis. The hospital outpatient department (OPD) case sheet record of each patient was obtained and analysed for brief history including the nature and duration of disease onset, presence of shortness of breath (New York Heart Association Classification (NYHA) Class), palpitation and its nature, diaphoresis, or exercise intolerance. Demographic details and clinical profiles of each patient were obtained from the OPD case sheet record. Baseline ECG and echocardiography parameters of each patient were also obtained from the OPD case sheet record of each patient. ECG was analysed for the rhythm, and individual echocardiography report was analysed for definite aetiology and the presence of thrombus in the left atrial appendage or left ventricle. Data were entered in Microsoft Excel software 2021 (Microsoft Company, Redmond, Washington, USA) in tabular form. All nominal and ordinal variables were represented in tabular form, and descriptive statistics was used to analyze the data in SPSS software (version 21) (SPSS Inc, Chicago, USA).

## Results

Table 1 reflects the baseline parameters of the study subjects. We analysed 117 patients with non-valvular atrial fibrillation less than 40 years of age between May 2020 and 2022. The mean age of our study population was 33.6±5.8 years. Most of the study population was between 30 and 40 years (80%), and the very young population (i.e., < 30 years) constituted 20% of the study population. Males constituted the majority of the study population (75%) as compared to females as atrial fibrillation is known to be predominant in males. Smokers constituted 18% of the study population, few patients (7%) had baseline dyslipidaemia, and only one patient had a family history of premature sudden cardiac death.

**Table 1:** Baseline Variables of Study Subjects

Variables	Total number of patients (%) n=117
Age (mean±SD) in years	33.6±5.8
< 30 years	94 (80)
30-40 years	23 (20)
Male	88 (75)
Female	29 (25)
Obesity	9 (8)
Alcohol intake	18 (15)
Smoking	21 (18)
Dyslipidaemia	8 (7)
Family history of sudden cardiac death	1 (1)

**Figure 1:**
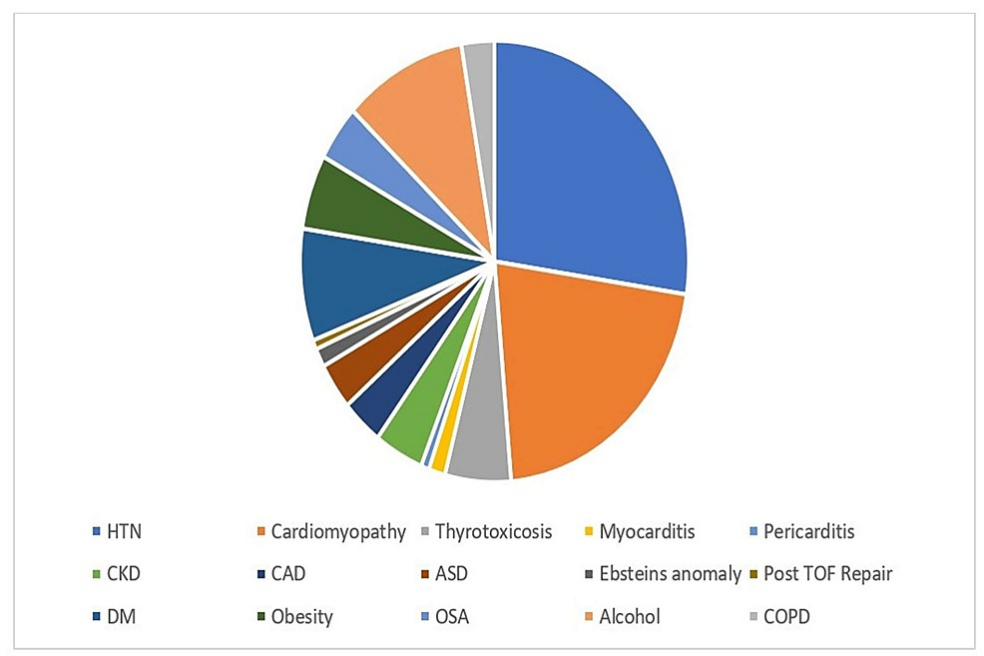
Aetiology of Atrial Fibrillation in Young Adult Population CKD: chronic kidney disease, CAD: coronary artery disease, OSA: obstructive sleep apnoea, COPD: chronic obstructive pulmonary disease, HTN: hypertension, ASD: atrial septal defect, TOF: tetralogy of Fallot, DM: diabetes mellitus.

Table 2 and Figure 1 reflect the burden of risk factors behind non-valvular atrial fibrillation in the young population. Hypertension was the commonest risk factor behind early-onset non-valvular atrial fibrillation in the young (40%) followed by dilated cardiomyopathy including both non-ischaemic and ischaemic cardiomyopathy (25%). Thyrotoxicosis (8%), obesity (8%), obstructive sleep apnoea (6%), and chronic kidney disease (5%) constituted a minority of cases among young patients with non-valvular atrial fibrillation. The commonest social factor resulting in non-valvular atrial fibrillation was an intake of alcohol of more than one drink per day (more than 12 g of pure alcohol) (15%). Among sick patients visiting the outpatient department, myocarditis in children (2%) was associated with atrial fibrillation. Hypokalaemia triggered atrial fibrillation in 2% of cases who visited the outpatient department for tachyarrhythmia. Acute pericarditis in young was associated with atrial fibrillation. Hypertrophic cardiomyopathy in the young (4.6%) constituted a minority among young patients with non-valvular atrial fibrillation. Two patients with peripartum cardiomyopathy presented with atrial fibrillation. Five patients with atrial septal defect had atrial fibrillation and were approaching the age of 40 years. Two patients with Ebstein's anomaly presented with atrial fibrillation, and one patient with post-tetralogy of Fallot total correction presented with atrial fibrillation.

**Table 2:** Aetiology of Non-Valvular Atrial Fibrillation in the Young CKD: chronic kidney disease, MI: myocardial infarction.

Risk factor		Total number of patients (%)
Hypertension		47 (40)
	Renal artery stenosis	28 (24)
Cardiomyopathy	Non-ischemic cardiomyopathy (NICM)	20 (17)
	Ischemic cardiomyopathy	9 (7.6)
	Hypertrophic cardiomyopathy	5 (4.6)
	Peripartum cardiomyopathy	2 (1.7)
Thyrotoxicosis		9 (8)
Obstructive sleep apnoea (OSA)		7 (6)
Myocarditis		2 (1.7)
Pericarditis		1 (0.85)
CKD		6 (5)
Hypokalaemia		2 (1.7)
Coronary artery disease (CAD)		5 (4)
	Inferior wall MI	3 (2.56)
	Anterior wall MI	2 (1.70)
Diabetes mellitus		14 (12)
Bronchial asthma		5 (4)
Congenital heart disease	Atrial septal defect	5 (4.27)
	Ebstein's anomaly	2 (1.70)
	Post-tetralogy surgical repair	1 (0.85)

Table 3 summarises the clinical, serology, ECG, echocardiography, and management of young patients with non-valvular atrial fibrillation. Most of the young patients had paroxysmal atrial fibrillation (70%), and 30% of them had persistent or permanent atrial fibrillation requiring direct current (DC) cardioversion and anticoagulation, respectively. Renal artery stenosis was present in 60% of patients with hypertension, and pheochromocytoma was diagnosed in only one young case of hypertension. Most patients presented with episodic palpitation (90%). Most patients (60%) were having shortness of breath in NYHA Class II, and 10% of cases were in NYHA Class III in whom fast ventricular rate was contributing towards shortness of breath. Seventeen per cent of cases complained of intermittent diaphoresis during the episode of atrial fibrillation. Coronary artery diseases constituted 4% of cases, and 4% of cases elicited a history of cerebrovascular accident. Diabetes was detected in 12% of cases where episodic hypoglycaemia may have contributed to the development of paroxysmal atrial fibrillation. Forty per cent of young patients with non-valvular atrial fibrillation were in heart failure with raised N-terminal pro-brain natriuretic peptide (NT-proBNP) (> 125 pg/ml). Bronchial asthma was an associated disease in 4% of young cases with non-valvular atrial fibrillation, the aetiopathogenesis of which is unknown. Our study presents an insight into the development of non-valvular atrial fibrillation in the young where left ventricular insults with two forms, i.e., either hypertension or left ventricular systolic dysfunction, were the two triggering factors towards the development of atrial fibrillation in the young secondary to an acute rise in left atrial pressure secondary to increase in left ventricular diastolic pressure which prompted anomalous left atrial pulmonary vein firing. Most patients were managed conservatively (84%) with rate-controlling agents, and three patients had undergone pulmonary vein isolation for the same. Our study provides new insight that young patients with hypertension and left ventricular systolic dysfunction should be looked at for the development of atrial fibrillation. Holter analysis was used in 14% of cases to record atrial fibrillation. One young patient with diabetes mellitus with atrial fibrillation had a large floating thrombus in the left ventricle in whom the aetiology of the thrombus in the left ventricle was obesity. Transthoracic or transoesophageal echocardiography delineated left atrial appendage thrombus in only 17% of cases, and most cases (86%) of those were having left ventricular systolic dysfunction. Anticoagulation (novel anticoagulants (NOACS)) was advised according to the congestive heart failure, age, diabetes mellitus, stroke, vascular disease, sex (CHADS2 VASC) score. The rate control strategy was adopted in most of the patients (84%), whereas the rhythm control strategy (14%) was adopted in rest, especially in patients with fast ventricular rate and left ventricular systolic dysfunction.

**Table 3:** Clinical, Serology, ECG, Echo Parameters, and Management of Patients With Young Non-Valvular Atrial Fibrillation NYHA: New York Heart Association Classification, NT-proBNP: N-terminal pro-brain natriuretic peptide.

Study variables		Number (percentage) N=117
Nature of atrial fibrillation	Paroxysmal	82 (70)
	Persistent or permanent	35 (30)
NYHA Class	NYHA Class II	70 (60)
	NYHA Class III	12 (10)
	NYHA Class IV	6 (5)
Episodic palpitation		101 (90)
Diaphoresis		20 (17)
History of cerebrovascular accident		5 (4)
Serum NT-proBNP > 125 pg/ml		47 (40)
Atrial fibrillation in ECG		101 (86)
Atrial fibrillation in Holter analysis		16 (14)
Left atrial appendage thrombus in echocardiography		20 (17)
Left ventricular thrombus in echocardiography		1 (1)
Management strategy	Rate control strategy	98 (84)
	Rhythm control strategy	16 (14)
	Pulmonary vein isolation (PVI)	3 (2)

## Discussion

Non-valvular atrial fibrillation in the young population comprises an important clinical entity. Fibrotic atrial remodelling, breaking down of atrial impulses into small wavelets, non-homogeneous conduction of atrial impulses across the atrium, and anomalous firing of pulmonary veins result in atrial fibrillation in the young population. Early recognition of atrial fibrillation in the young population can prevent them from myriad and devastating complications like embolic central nervous system stroke and peripheral stroke. We tried to delineate the aetiology behind young patients with atrial fibrillations, stratified them according to the clinical profile, and managed those patients with a proper anticoagulation regimen to prevent embolic stroke. We adopted the classification given in the article by Horng et al. of age groups to define young adults who are less than 40 years of age [4].

Our study demonstrated that non-valvular atrial fibrillation was more common after the third decade among the young adult population as atrial compliance starts failing after the third decade of life. At a relatively younger age, atrial fibrillation is quite uncommon as atrial compliance is quite good and the atrium does not suffer acute stretch even if it faces an acute rise in the atrial pressure and conduction is also homogenous. Males constituted the majority of the study population as it is known that atrial fibrillation is a male predominant disease. Atrial fibrillation is more common in males due to a shorter atrial refractory period and larger left atrial size favouring re-entry and atrial fibrillation. Atrial fibrillation is divided into two types: adrenergic atrial fibrillation and vagotonic atrial fibrillation. As males often carry out outdoor activities in India, they suffer from exercise-induced adrenergic atrial fibrillation more as compared to females.

The most common aetiology behind non-valvular atrial fibrillation in young adults was hypertension. The association between hypertension and atrial fibrillation is well established. Hypertensive patients carry a 14% additional risk of developing atrial fibrillation [5], and the presence of hypertension increases the risk of developing atrial fibrillation by 1.42-fold [6]. Hypertension produces concentric ventricular remodelling and a subsequent increase in left ventricular end-diastolic pressure which consequently elevates the left atrial pressure. An increase in left atrial pressure triggers pulmonary vein firing which induces atrial fibrillation, and fibrotic left atrial remodelling starts which facilitates inhomogeneous conduction across the atrium breaking the impulses into wavelets and precipitating atrial fibrillation and degenerating at a faster rate. Renal artery stenosis was the commonest cause behind hypertension in young adults; electrolyte imbalance in advanced renal artery stenosis also contributes towards the development and perpetuation of atrial fibrillation among the young population. One patient had pheochromocytoma in whom atrial fibrillation was adrenergic atrial fibrillation as the pouring of sympathomimetic amines into the systemic circulation was the triggering factor behind the abnormal and asynchronous firing of the pulmonary veins leading to atrial fibrillation.

The second most common cause of non-valvular atrial fibrillation in young adults was dilated cardiomyopathy in whom 55.56% were non-ischemic dilated cardiomyopathy (NICM). Rest were ischemic cardiomyopathy in the young who suffered a myocardial infarction long back, and the culprit artery was not revascularised leading to cardiomyopathy. About 30%-40% of patients with left ventricular systolic dysfunction develop atrial fibrillation in due course of time [7-9]. The mechanism of atrial fibrillation in NICM is a rise in left ventricular end-diastolic pressure secondary to left ventricular systolic dysfunction which in turn increases the left atrial pressure which triggers the pulmonary vein firing and left atrial fibrotic remodelling facilitating inhomogeneous conduction. The mechanism of atrial fibrillation in ischemic cardiomyopathy is old atrial infarct and subsequent remodelling which may be secondary to occlusion of the right coronary artery resulting in right atrial infarct or occlusion of the left atrial branch of the left circumflex artery leading to left atrial ischemia, infarct, and atrial fibrillation. The mechanism of atrial fibrillation when the left anterior descending coronary artery is the culprit artery is an acute rise in left ventricular end-diastolic pressure which in turn rises the left atrial pressure leading to atrial fibrillation which most often responds to digoxin.

The commonest social factor behind the development of non-valvular atrial fibrillation in young (15%) was alcohol intake or binge drinking. The association between alcohol intake and atrial fibrillation is well-established [10]. Alcohol intake induced a sympathetic surge in circulation and its toxic metabolite acetaldehyde irritates the atrium to fire abnormally resulting in atrial fibrillation. In India, “moon shine drinkers” also constitute a major part as they drink alcohol the whole night and develop atrial fibrillation the next day morning which is known as “Holiday Heart Syndrome or Saturday Night Syndrome” in old literature.

Thyrotoxicosis induced atrial fibrillation by exaggerating sympathetic drive and inducing adrenergic atrial fibrillation. About 8.3% of thyrocardiac patients develop atrial fibrillation as the disease progresses [11,12]. Both hypothyroidism and hyperthyroidism induce atrial fibrillation in young and middle-aged adults. In our study, 8% of patients were thyrocardiac secondary to either Hashimoto thyroiditis or multinodular goitre. Those patients were well managed with propranolol 80 mg per day which either converted them to sinus rhythm or controlled the ventricular rate.

Five per cent of our patients were obese where obesity was the cause of atrial fibrillation emphasising the deleterious effect of lipotoxicity on the heart. The presence of a large amount of pericardial fat contributes to the development of atrial fibrillation by producing inflammation [13]. Obese patients usually have a wide curtain of fat around the heart which derange the electromechanical homeostasis of the atrium leading to atrial fibrillation in the young population. Atrial fibrillation in the obese is a good example of lipotoxicity on the heart by the presence of abundant adipose tissue around the heart producing inflammation, especially in abdominal obesity. Obstructive sleep apnoea induces atrial fibrillation by producing a neurohormonal imbalance in the atrium besides producing abnormal sympathetic surge at night. Autonomic dysregulation, sympathetic surge, oxidative stress, endothelial dysfunction, and left atrial stretch contribute to the development of atrial fibrillation in obstructive sleep apnoea [14,15].

Chronic kidney disease (CKD) produces atrial fibrillation by producing electrolyte imbalance in the form of hypokalaemia, hyponatraemia, hypomagnesemia, and hypocalcaemia. Altered intracellular electrolyte hemostasis induced abnormal atrial firing and atrial fibrillation. The risk of developing atrial fibrillation in CKD patients increases directly with the risk of decreasing glomerular filtration rate (GFR) [16]. The association between myocarditis [17] and pericarditis causing atrial fibrillation is also known today [18]. Myocarditis (2%) and acute pericarditis (1%) resulted in atrial fibrillation in very young persons by irritating the atrial myocytes as there remains a curtain of pericardium over both the atria; inflammation of the overlying pericardium irritates the neighbour atrial myocytes.

Nine per cent of young adults with atrial fibrillation were secondary to congenital heart disease out of which atrial septal defect was the commonest. Atrial fibrillation occurs in approximately 20% of patients with atrial septal defect [19]. It also occurs in Ebstein anomaly [20] and post-surgical correction of the ventricular septal defect, tetralogy of Fallot, pulmonary stenosis, and transposition of great arteries. A decrease in atrial compliance with an increase in left ventricular stiffness with age was the mechanism behind atrial fibrillation in ASD. Two patients with atrial fibrillation had Ebstein anomaly where the large aneurysmal right atrium was firing to produce atrial fibrillation. One patient with post-tetralogy of Fallot (TOF) repair had atrial fibrillation in whom the right atriotomy scar was responsible for inhomogeneous conduction and subsequent atrial fibrillation.

Most patients in our study had paroxysmal atrial fibrillation as in a young heart the sinus node dominates the rhythm most often and fibre inhomogeneity is not there in the young heart to produce inhomogeneous conduction and atrial fibrillation. There exists a dictum in the treatment of atrial fibrillation: “Every atrial fibrillation should be provided one chance to return to sinus rhythm by either mechanical or chemical cardioversion”. We opted for cardioversion in all patients with permanent atrial fibrillation. Most of the young patients with atrial fibrillation were symptomatic (90%) with raised NT-proBNP (40% of cases). Atrial fibrillation in young most often presents with a fast ventricular rate. During the episode of atrial fibrillation, both atria behave like a lake full of water, and passive congestion in the pulmonary veins gives rise to dyspnoea.

Acute coronary artery disease was present in 4% of cases where 3% were having inferior wall myocardial infarction having lesions in the right coronary and left circumflex artery and only 1% was having anterior wall myocardial infarction. The atrial fibrillation in inferior wall myocardial infarction was transient in nature and it responded to beta blockers. The atrial fibrillation in anterior wall myocardial infarction was non-responsive to beta blocker and needed chemical cardioversion with amiodarone. Atrial fibrillation occurs in 6%-10% of cases with acute myocardial infarction [21] due to ischemia-induced abnormal calcium handling causing delayed depolarisation and abnormal firing of atrial myocytes.

Twelve per cent of cases in our study had diabetes mellitus. Diabetes mellitus is an independent risk factor behind atrial fibrillation by producing fibrotic atrial remodelling through advanced glycosylation end products [22,23]. Episodes of both hypoglycaemia and hyperglycaemia induce atrial fibrillation. There was a casual association of bronchial asthma with atrial fibrillation in 4% of cases. There is a rare description of the association between chronic obstructive pulmonary disease (COPD) and atrial fibrillation [24].

Five patients with hypertrophic cardiomyopathy developed atrial fibrillation. Atrial fibrillation is the most common arrhythmia in hypertrophic cardiomyopathy. Atrial fibrillation occurs in 10%-28% of patients with hypertrophic cardiomyopathy [25] secondary to atrial fibrosis due to increased left atrial pressure secondary to diastolic dysfunction and mitral regurgitation [26]. Peripartum cardiomyopathy is also associated with atrial fibrillation [27] as noted in our study.

Atrial fibrillation is usually considered an elderly phenomenon among physicians and the general population. Non-recognising atrial fibrillation in the young population leads to cryptogenic stroke in the young with associated morbidity and mortality. Our study presents a new insight into the clinical-aetiological profile and spectrum of non-valvular atrial fibrillation in the young adult population. Early scrutiny and management of those cohorts of patients can prevent the population from the morbid consequences of atrial fibrillation.

## Conclusions

The present study provides insight into the profile and spectrum of non-valvular atrial fibrillation in relatively young populations contrary to the belief that atrial fibrillation is an elderly phenomenon. Hypertension and cardiomyopathy with left ventricular systolic dysfunction contribute towards the development of non-valvular atrial fibrillation in the majority of young adults followed by unrecognised congenital heart diseases, thyrotoxicosis, obesity, and obstructive sleep apnoea. Myopericarditis, chronic kidney disease, dyselectronemia, young-onset diabetes mellitus, and chronic obstructive pulmonary disease also contribute to the development of non-valvular atrial fibrillation among young adults. Atrial fibrillation among young adults makes them symptomatic early, and early therapeutic anticoagulation mitigates the risk of future embolisation by having a thrombus in the left atrial appendage or left ventricle. Careful measurement of blood pressure and careful assessment of left ventricular systolic dysfunction with proper therapeutic measures in young adults can save them from the morbidity and mortality of early-onset non-valvular atrial fibrillation.
